# Lived experiences of radiology caregivers during a health crisis: A COVID-19 case analysis

**DOI:** 10.4102/hsag.v29i0.2532

**Published:** 2024-07-08

**Authors:** Shillah N. Hundah, Maureen N. Sibiya, Thandokuhle E. Khoza

**Affiliations:** 1 Department of Radiography, Faculty of Health Sciences, Durban University of Technology, Durban, South Africa; 2 Division of Research, Innovation and Engagement, Mangosuthu University of Technology, Umlazi Campus, Durban, South Africa

**Keywords:** health crisis, COVID-19, frontline radiology caregivers, radiographers, radiologists, experiences, challenges, eThekwini

## Abstract

**Background:**

Health crises have been linked with the exacerbation of pre-existing difficulties and the emergence of unique challenges, as evidenced by the impact of coronavirus disease 2019 (COVID-19) on health caregivers worldwide. Baseline data allow for reflection and preparation for any future health emergencies therefore giving impetus to phenomenological enquiries among the experiencers.

**Aim:**

This study aimed to explore the lived experiences of the eThekwini district frontline radiology caregivers during the COVID-19 pandemic.

**Setting:**

The study was conducted in public and private radiology departments in the eThekwini district of KwaZulu-Natal, South Africa.

**Methods:**

A qualitative, interpretative phenomenological analysis methodology was adopted in a multi-method data-collection context comprising semi-structured interviews and focus group discussions (FGDs) among 24 radiologists, radiographers, and radiology nurses obtained by non-probability sampling. Data were transcribed verbatim and analysed using an interpretative phenomenological approach.

**Results:**

Three superordinate themes emerged, namely: (1) duties and roles during the COVID-19 pandemic, (2) work-related challenges, (3) personal challenges.

**Conclusion:**

Frontline radiology caregivers experienced increased workload, staff shortages, salary cuts, personal protective equipment (PPE) shortages, non-recognition, poor managerial support, disrupted social relations, and poor work–life balance. This necessitates the need for the radiology departments to address staffing, infection prevention and control deficits, and invest in support interventions to assist frontline radiology caregivers during health crises.

**Contribution:**

The findings comprise baseline information that can be used for reflection and guiding radiology departments in preparing for any future health crises.

## Introduction

Frontline health caregivers are often exposed to nosocomial infections that can be potentially exacerbated by health crises, as reportedly healthcare workers constituted 20% and 10% of the population infected with Severe Acute Respiratory Syndrome Coronavirus (SARS) 1 epidemic and the novel coronavirus disease 2019 (COVID-19), respectively (Mossburg et al. 2019:1; Liu et al. 2012:15; World Health Organization [WHO] 2020). Spreading through air droplets and contaminated surfaces, the highly infectious novel coronavirus, SARS-CoV-2, was first reported in Wuhan, China in 2019 and hit South African shores on 05 March 2020. As of August 2020, over 27 000 healthcare workers had been infected with COVID-19 while 240 had succumbed to it, with the KwaZulu-Natal (KZN) province also suffering significant infection rates (Centres for Disease Control and Prevention [CDC] 2023; Ramphul, Mejias & Ramphul 2020:274; Singh 2020; South African Department of Health [NDoH] 2020:1).

In addition to infections and deaths, several consequences such as economic, educational disruptions, unemployment, and mental impacts were recorded worldwide (Sekyere et al. 2020). Shortage of hospital beds and Personal Protective Equipment (PPE) were among the difficulties faced by the South African healthcare industry and like any other frontline health workers, the radiology and radiography staff were also exposed to the highly infectious respiratory disease COVID-19 (Singh 2020; Zanardo et al. 2020:265; WHO 2020). Moreover, the increased use and indispensability of the chest radiograph (CXR) and the computed tomography (CT) scan in the management of COVID-19 patients for differential diagnosis and diagnosis of COVID-19 complications, coupled with reported shortages of infection prevention and control (IPC) equipment and protocols could potentially aggravate their risk of infection within the workplace (Murphy et al. 2022:388; Zanardo et al. 2020:265).

Studies on the COVID-19 experiences of healthcare workers and radiographers in various geographical locations including South Africa have commonly reported increased workload, shortage of PPE, and mental health repercussions (Akudjedu et al. 2021:1219; Murphy et al. 2022:384; Lewis & Mulla 2020:346; Van de Venter et al. 2021:593; Watermeyer, Madonsela & Beukes 2023:2). The highlighted experiences have exposed lessons learned and areas of improvement within these specific locations. However, the unique lived experiences of radiologists, radiographers, and radiology nurses within the private and public radiology departments in the eThekwini district of KZN, South Africa, have not been explored, thereby preventing the reflective process, addressing areas in need of attention and planning for any further health crises.

Despite collectively being in the health sector, findings of lived experiences of a phenomenon cannot be generalised because of the diversity of the statistics and repercussions of COVID-19 in different geographical, social, and economic environments (Worldometer 2020). Nationally, the South African public healthcare system is decentralised at the provincial level; hence, provinces have different economic and operational statuses with records of non-operational or faulty radiology equipment and deteriorating healthcare services reported in KZN as well as unique occupational stressors among the KZN radiographers (Gam, Naidoo & Puckree 2015:18; Modisakeng et al. 2020:3). Therefore, the consequences and impact of COVID-19 could have been different with possible differing experiences of the frontline radiology caregivers, necessitating the need to capture their own phenomenological voice. This study therefore intended to explore the lived experiences of radiologists, radiographers, and radiology nurses working in private and public radiology departments within the eThekwini district of KwaZulu-Natal, South Africa during the COVID-19 pandemic to provide baseline information to necessitate reflective practice and preparation of radiology departments for any future health crises.

## Research methods and design

### Research design

Taking a constructivist epistemological position and interpretivist theoretical stance, the qualitative, interpretative phenomenological analysis (IPA) approach was adopted to allow for a detailed exploration and sensemaking of each individual participant’s lived experience during the COVID-19 pandemic (Smith, Flowers & Larkin 2022:1; Moon & Blackman 2014:3).

### Setting

The study was conducted in the eThekwini district of KZN province, South Africa within both private and public radiology departments. The public radiology departments included in the study were from diverse health facilities comprising central, tertiary, regional, and district hospitals as well as community health centres allowing for a critical exploration of the lived experiences of the radiology caregivers in various radiology settings.

### Study population and sampling strategy

The population comprised Health Professions Council of South Africa (HPCSA) registered radiologists and diagnostic radiographers as well as the South African Nursing Council (SANC) registered radiology nurses working in private and public eThekwini district radiology departments to ensure homogeneity of the sample (Pietkiewicz & Smith 2014:9). Eight public radiology departments and three private radiology practices with at least 11 branches within the eThekwini district were included in the study as they constituted the target population. The inclusion criteria comprised all frontline radiology caregivers with a minimum of 2 years’ service at their respective hospitals to exclude the potential impact of lack of work experience on participants’ lived experiences. Participants needed to be registered with HPCSA within either the independent, private, specialised or sub-specialised practice categories or SANC within either the enrolled or registered nurse categories. Community service nurses, radiology registrars, community service radiographers, student radiographers, and radiographers not working in facilities attending to COVID-19-related cases were excluded from the study as their lived experiences could have potentially been impacted by several other external factors not related to the radiology department.

The maximum variation purposive sampling method was adopted to allow for the capturing of a wider range of perspectives and representation of each professional category from each of the radiology settings (Johnson & Christensen 2014). The population was first categorised according to their working sectors (public and private), and then professional categories (radiologists, radiographers, and radiology nurses). In keeping with phenomenological studies that enable the researcher to generate and analyse data in detail with an utmost number of 15 participants, a minimum of four and a maximum of six participants from each professional category were interviewed (Pietkiewicz & Smith 2014:9). Consistent with Nyumba et al. (2018:23)’s recommendation that allows capturing of individual voices, a minimum of five and a maximum of eight participants were recruited for each of the two focus groups. The sample size was considered on a data saturation basis within the professional categories.

### Data generation

After ethics and gatekeeper permissions were granted, the interview and FGD guides were pre-tested by the researcher among participants who did not take part in the final data generation. Because of COVID-19 regulations, data were generated virtually and telephonically between May and September 2021 using a multi-method approach comprising semi-structured one-on-one interviews with 13 participants in the first phase (*n* = 13) and two FGDs (*n* = 2) constituting five and six participants each in the second phase. This allowed for the simultaneous capture of individual perceptions of eThekwini-based frontline radiology caregivers’ experiences as well as the ability to discuss work-related issues that individuals might fear to share in fear of victimisation (eds. Bauer & Gaskell 2000:48). Interview invitations were circulated among the population through their heads of departments (HODs). The invitations included information advising individuals to express their interest in participating in the study by either informing their HODs or emailing the researcher directly. The pre-tested interview and FGD guides comprised open-ended questions (Box 1), which were developed by interlinking the research study’s aim and research questions to enable a comprehensive exploration of the frontline radiographers’ experiences.

BOX 1 Interview and focus group discussion questions.

**Please describe how it has been working in your current job during the COVID-19 crisis.** (Interpersonal relationships, safety in the workplace, support from peers and support from management).

**Please describe your feelings around your safety (risk of infection) in the workplace.**

What factors (within the department or work environment) are influencing these feelings?

**How have your professional duties been impacted by working during the COVID-19 crisis?**

What factors are influencing this impact?

Who do you think is responsible for each mentioned factor?

COVID-19, coronavirus disease 2019.

### Measures of trustworthiness

Trustworthiness of the study was adopted throughout the study by incorporating the trustworthiness framework by Guba and Lincoln (Johnson & Christensen 2014). To ensure credibility of the study, the researcher developed an interview schedule and conducted a pilot study to sharpen their interviewing and group discussion facilitating skills. Peer validation and cross-checking of interview or FGD notes with audio recordings was conducted by the researcher and verified by the research supervisors (Johnson & Christensen 2014). As a form of an audit trail, a Microsoft OneNote research journal comprising interview and FGD schedules, notes, audio recordings, and reflective log was kept throughout the research process to establish dependability of the research study and outcomes. To ensure confirmability of the study’s findings, investigator triangulation was adopted by incorporating an independent data analyst and implementation of reflective journaling through use of the developed reflective log. Transferability of the study findings was established by adopting the maximum variation purposive sampling technique and provision of direct quotations from the participants within the results section (Creswell 2014; Denzin 2013:15; Johnson & Christensen 2014).

### Data analysis

**FIGURE 1:**
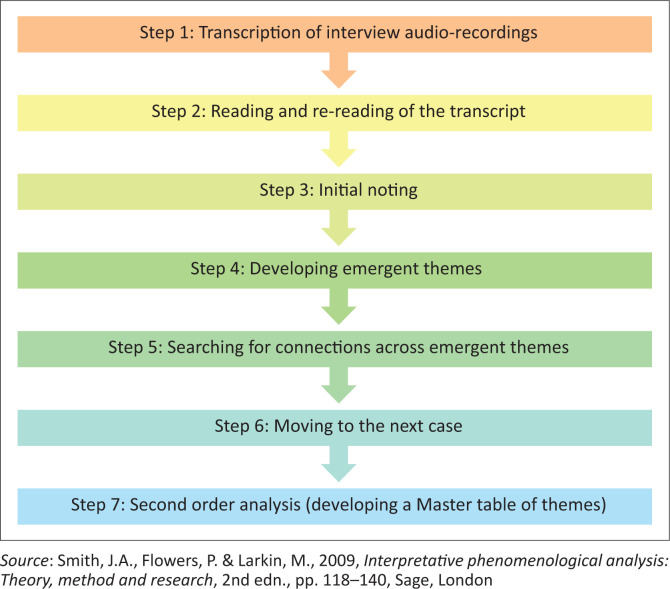
Adapted interpretative phenomenological analysis framework.

The interview and FGDs, which were audio recorded upon the participants’ approval, were transcribed into text data by an independent professional transcriber and then cleaned and verified by the researcher. After this the data were coded and categorised into themes through the use of the IPA; therefore, allowing the researcher to engross in the data. In line with double hermeneutics, this enabled the researcher to simultaneously capture the frontline radiology caregivers’ sense-making of their experiences and make sense of the shared accounts (Pietkiewicz & Smith 2014:11). The steps in Figure 1 were incorporated to allow a detailed case-by-case analysis of the interviews, FGDs, and capturing of individual voices and experiences during steps one to five; therefore, enabling the idiographic focus of IPA (Smith, Flowers & Larkin 2022:125). During the first five steps, the interview and FGD transcripts were individually read and re-read for better understanding (Smith & Osborn 2007:67). Any arising thoughts and outcomes of the exploratory analysis of semantic and language use in the transcripts were documented using a reflective log. During step four, common themes were checked for connections and grouped according to abstraction, subsumption, polarisation, contextualisation, numeration, and function (Smith, Flowers & Larkin 2022:130–137). A master table of themes for the sample was developed by second-order analysis, which entails a search across individual cases (Smith & Osborn 2007:75) (Step seven). To confirm the themes, an independent data analyst perused the transcripts and data analysis process.

### Ethical considerations

Prior to data collection, ethical approval (IREC 177/20) was obtained from the Durban University of Technology Institutional Research Ethics Committee and gatekeeper permissions obtained from the KZN department of health, eThekwini Health district, hospital and radiology departments’ management. Participants were provided with a letter of information and requested to provide written informed consent by completing the issued consent form prior to the interview or focus group discussion (FGD). The letter of information informed participants of the objectives, voluntary nature of the study, non-involvement of financial benefits, and their right to withdraw from the study at any time they felt uncomfortable with participating in the study. It also emphasised the prioritisation of confidentiality and anonymity during the study. In keeping with this, a separate demographics questionnaire was completed prior to data generation and each participant was allocated an anonymous code. Furthermore, no personal details were included on the interview or FGD transcripts. Participants were also provided with a form to indicate their preferred data-generation method (interview or FGD) and preferred platform (online or telephonic) without coercion. To address the challenge of bringing together participants for FGDs because of differences in work shifts and preferred data-collection times, an online poll site was used to reach a common time slot. During data generation, the researcher regularly observed participants for any signs of distress, and allowed the participants to take a break, stop the interview, or withdraw from the FGD if a need arose.

## Results

### Demographic characteristics

Most of the participants were working in public radiology departments, identified as female, and were in the age range 18 years – 49 years (Table 1). Grade 1–3 radiographers made up most of the sample, which also comprised four chief radiographers, two radiography managers, two radiology nurses, and four radiologists. The majority of the participants had 6 years – 10 years’ work experience within their roles, with the most experienced participants having more than 20 years. Many of the participants attended to COVID-19 patients with some attending to more than twenty COVID-19 patients per week.

**TABLE 1:** Participants’ demographic data.

Characteristics	Categories	Count (*n*)	%
Biological sex	Male	6	25.0
	Female	18	75.0
Age (years)	18–29	8	33.3
	30–39	8	33.3
	40–49	7	29.2
	50–59	1	4.2
Professional title	Enrolled and staff nurse	2	8.3
	Grade 1–3 Radiographer	12	50.0
	Chief Radiographer	4	16.7
	Radiography manager	2	8.3
	Radiologist	3	12.5
	Clinical Head of radiology unit	1	4.2
Duration in the role (years)	2–5	7	29.2
	6–10	11	45.8
	11–20	4	16.7
	20 and above	2	8.3
Working environment	Public	16	66.7
	Private	8	33.3
Number of COVID-19 patients attended to per week	None	3	12.5
	1–10	10	41.7
	11–20	7	29.2
	more than 20	4	16.7

COVID-19, coronavirus disease 2019.

## Themes

A detailed insight into the experiences of eThekwini-district based frontline radiology caregivers during the COVID-19 pandemic presented with three superordinate themes, namely: (1) duties and roles during the COVID-19 pandemic, (2) work-related challenges, (3) personal challenges (Table 2).

**TABLE 2:** Master table of superordinate and subordinate themes.

Superordinate themes	Subordinate themes	Key words and issues
Duties and roles during the COVID-19 pandemic	Use of radiology modalities in the management of COVID-19 patients	Projectional radiography, CT, ultrasound and mobile radiographic examinations
	Role of radiologists	Clinical duties and patient care, radiology reporting, consultation, screening of patients, justification of radiology requests and IPC.
	Role of radiographers	HPCSA scope of practice, IPC, management, screening x-ray request forms, patient care and interaction and administrative duties.
	Role of radiology nurses	IPC, clinical duties and patient care.
Work-related challenges	Unpreparedness and perceived increased risk of infection	PPE availability, role of management during the pandemic, management’s concern about the safety of employees, structural deficiency of radiology departments, increase in workload, adhering to strict IPC guidelines, inadequate PPE and clinicians not sharing enough patient information.
	Adaptation to changes in systems, protocols and structures in response to the pandemic	Booking of patients, duties and shift rotation, introduction of new cleaning protocols, use of PPE and social distancing in the workplace.
	Information about COVID-19	Inadequate information on COVID-19 transmission, scant information on the nature of the disease and poor interdepartmental information sharing when caring for person under investigation (PUI).
	Disruption of social relations because of social distancing	Disruption of peer-to-peer interactions, limit on number of people in a closed space at a time, and cessation of social events.
Personal challenges	Physical health	Burnout because of increased workload, back pain from carrying cassettes to the ward when doing BSUs, contracting COVID-19, fatigue as an after effect of contracting COVID-19 and other health-related issues.
	Financial hardships	Salary reductions and paying out of pocket for COVID-19 related services.
	Social impact	Social shielding responsibility for family members and strained interactions with family members.

COVID-19, coronavirus disease 2019; CT, computed tomography; IPC, infection prevention and control; HPCSA, health professions council of South Africa; PPE, personal protective equipment; BSU, bedside unit; PUI, Person under Investigation.

### Superordinate theme 1: Duties and roles during the coronavirus disease 2019 pandemic

Duties and roles during the COVID-19 pandemic comprised four subordinate themes, namely: (1) use of radiology modalities in the management of COVID-19 patients, (2) role of radiologists, (3) role of radiographers, (4) role of radiology nurses. Participants outlined that there was an increased demand for projectional radiography and CT scans, specifically mobile chest radiographic examinations within dedicated COVID-19 wards as well as computed tomography pulmonary angiograms (CTPAs) for patients during both active and post-COVID-19 phases. Ultrasound, CT brain, and high-resolution computed tomography (HRCT) were also conducted:

‘[*W*]e do a lot of CTs for COVID-19 patients. It is mostly chest, followed by brain … other emerging findings maybe on x-rays or ultrasound … we do CTPAs to look for pulmonary embolism [*PE*] … if they find something on ultrasound, those patients end up in CT scan …’ (Radiologist, Male, Participant 1)

Frontline radiology caregivers’ duties and roles expanded and differed among professions with the commonalities of IPC responsibilities, patient care, and clinical duties aligning with HPCSA and SANC-approved scope of practice:

‘[*W*]e worked with PPE, cleaned our machines thereafter … we isolated rooms as designated for positives or Person under Investigation [*PUI*]s, batched patients.’ (Radiologist, Female, Participant 6)

Radiographers were frequently in close contact with COVID-19 patients. In addition to screening x-ray request forms for COVID-19 status to ensure appropriate IPC measures were implemented, radiographers conducted clerical duties as well as managerial tasks for those who were in managerial positions:

‘While one is entering the details, the other radiographer has to position and another can take cassettes and process them … you will be helping each other to lift the patient …’ (Radiographer, Male, Participant 2)

‘[*B*]eing a radiography and venue manager, I have implemented tactical ways of how we could work around PPE shortage.’ (Radiographer, Female, Participant 11)

However, some radiographers in private practice performed extended duties such as administration of contrast media during CT examinations:

‘[*S*]ometimes, I would administer contrast media hand injections on night duty because I did not have anyone else on site. Our radiologist then agreed to take responsibility for it.’ (Radiographer, Female, Participant 10)

Radiology nurses supported patients with pre- and post-radiology examinations, although most COVID-19 patients were attended to by their accompanying COVID-19 ward nurses to minimise the spread of infection:

‘[*T*]he nursing sister would put PPE on and she would go in and check your line and check that we have made a successful intravenous [*IV*] injection.’ (Radiographer, Female, Participant 10)

‘I have to prepare patients for CT scans … and also either myself or the radiologist have to resuscitate patients in case of emergencies.’ (Radiology nurse, Female, Participant 8)

Radiologists continued with their clinical duties during radiology examinations as well as radiology reporting, consultation, screening, and justification of radiology requests, particularly for CT scans. Alongside this was ongoing in-house developmental training to develop expertise around the new infection, COVID-19:

‘[*W*]hen we are doing the continuous education in the department, we have to include COVID-19 … especially as the Head of Department, I have to make sure that the doctors [*radiologists*] are aware of all these manifestations of COVID-19.’ (Radiologist, Male, Participant 1)

### Superordinate theme 2: Work-related challenges

Work-related challenges faced by participants resulted in four subordinate themes namely: (1) Unpreparedness and perceived increased risk of infection, (2) adaptation to changes in systems, protocols and structures in response to the pandemic, (3) information about COVID-19, and (4) disruption of social relations because of social distancing. Participants based in public healthcare institutions never felt safe working in their departments as their perceived risk of contracting the virus was heightened owing to a plethora of issues such as unavailable or inadequate PPE particularly in institutions where radiography and radiology staff were not being considered frontline workers:

‘[*I*]t has been very stressful because of the lack of PPE specifically … they were telling us that it is the people who physically deal with the patients … but we do come in direct contact [*with patients*] …’ (Radiographer, Female, Participant 3)

‘[*T*]hey are saying we do not need most of these things – gloves, gowns, aprons.’ (Radiology nurse, Female, Participant 18)

Conversely, some participants perceived that the shortage of PPE was resultant from management’s reluctance to source adequate stocks, therefore, deliberately placing employees at risk of infection. As a result, some radiology caregivers resorted to purchasing their own PPE to ensure safety within the workplace. Others believed this was because of the ordering policy in government healthcare institutions:

‘[*W*]e are still not fully equipped with the equipment to efficiently protect ourselves and this is coming from their directive [*management*]. It comes from the people who do not even come into contact with COVID-19 patients. They do not even know how it feels and they do not even understand how important it is to us … they do not care and think whether what they are giving to us as PPE is adequate and suitable …’ (Radiographer, Female, Participant 21)

‘[*W*]e are using the ordering procedures where head of departments, [*Radiography Assistant Director*] can approve PPE purchase … but they must go through a channel of local managers … who do not know the type of job that we do.’ (Radiographer, Male, Participant 2)

Contrary to what was occurring in public radiology departments, management in most private radiology departments ensured staff received enough PPE:

‘I was very impressed with my place of practice. We were not limited on any number of PPEs.’ (Radiographer, Female, Participant 10)

Participants within public radiology departments perceived management as non-supportive and non-empathetic; hence, exacerbating the ongoing challenges:

‘There was no assistance or support obtained from management, during all of this (tested COVID-19 positive). I was asked if I am sure I cannot make it to work.’ (Radiographer, Male, Participant 7)

‘[*I*] must admit [*we do not get support from management*]. I am talking about outside the department … We were not triaged as significantly at risk, it was a little bit offensive …’ (Radiologist, Female, Participant 6)

Participants with existing co-morbidities felt that management’s lack of support and concern for employees’ well-being resulted in heightened risk of contracting the virus and poor access to the vaccine:

‘I did not think management appreciated the level of immunosuppression I was at. Accessibility to the vaccine was poor on our side, so we were hearing about other hospitals where everybody was vaccinated … and we were still waiting.’ (Radiologist, Female, Participant 6)

Staff shortages impacted service delivery:

‘When I showed symptoms and took COVID-19 leave, I was not at work for a good two weeks, and there was no one carrying on the work in the department, because I am the only one … the department was closed so, unfortunately, the x-ray patients had to go to x-ray departments in other hospitals …’ (Radiographer, Male, Participant 7)

‘I am the only nurse in the department, so I work straight shifts. During night shift, the radiographers contact the Accident and Emergency [*A&E*] department for any medical assistance.’ (Radiology nurse, Female, Participant 8)

Public radiology departments were not structurally sufficient for the implementation of COVID-19-related IPC protocols because of limited space and ventilation, thereby placing both staff and patients at an increased risk of infection. This was also coupled with the nature of radiography-related work:

‘[*T*]hey found that we do not have a good ventilation system in place … at some point in time you have 86 patients waiting in one waiting area. So, it becomes very challenging to adhere to social distancing directives … it is also not practical because in radiography we work in contact with patients … among colleagues … because radiography is a teamwork you cannot necessarily social distance.’ (Radiographer, Male, Participant 2)

Radiologists relayed similar concerns:

‘[*T*]he radiology department, was not geared for social distancing … Here you have doctors coming to look at radiology images and discuss cases in one room …’ (Radiologist, Male, Participant 13)

Despite this, participants tried their best to adhere to the recommended IPC protocols although at times they slackened because of fatigue:

‘[*B*]ut as far as possible, we are trying to be as cautious; hand hygiene, keeping our mask on all the time. We are trying our best.’ (Radiographer, Female, Participant 3)

‘[*I*] think everybody is at the stage where they are sort of fed up a little bit, and you maybe tend to let your guard down when you should not.’ (Radiographer, Female, Participant 9)

Radiology health caregivers found the process of donning and doffing PPE to be cumbersome, time consuming, and uncomfortable when they had to wear it for longer periods. Together with the cleaning of equipment between patients and after visiting the COVID-19 ward for mobile radiographic examinations, this increased workload and was perceived as overwhelming because of associated staff shortages:

‘[*W*]e cannot do as many patients as we would like to, because of cleaning time. It does increase the workload … If we had more staff, I do not think we would have been in the situation we were in … my call was coming around so quickly …’ (Radiographer, Female, Participant 10)

‘[*W*]earing masks all the time is exhausting. It is also stressful because some of us are reacting to these masks …’ (Radiology nurse, Female, Participant 18)

To accommodate the increase in demand for medical imaging and safety of staff and patients, CT scans were booked for the late afternoon after other patients had been attended to, unless it was urgent:

‘If it is a really urgent scan, sometimes we will try and squeeze it in where we have got a gap, but generally we do try and sort of wait a little bit later in the day … so that it does not create a huge impact on our list … because you have got to wait for the patient to come down, scan the patient and do a full clean of the room …’ (Radiographer, Female, Participant 9)

Changes in protocols, staff shortages, COVID-19 infection among staff, and sick leave severely impacted duties and shift rotation; therefore, radiography managers got more involved with clinical duties to relieve the pressure where possible:

‘[*P*]ublic hospitals are facing a shortage of staff … and also because we now started to see a lot of patients … then as managers we had to help with Bedside Unit [*BSU*]s and CT scans … so that we can ease the load on radiographers …’ (Radiographer, Female, Participant 4)

Social distancing protocols disrupted peer-to-peer interactions and brought social events to an end as only a limited number of people were allowed in a closed space at a time:

‘Our social events in the department, these are the things that keep us together as a team, were limited and there was change in the nature of those.’ (Radiologist, Female, Participant 6)

Early into the pandemic, there was scarcity of information on the nature, spread, and prevention of the virus. Also noted was a significant number of incomplete radiology request forms from referring clinicians, as they did not share adequate COVID-19-related information that was required to ensure IPC protocols for PUI or COVID-19 patients are implemented:

‘Especially in the beginning, there was not a lot of … guidelines about PPE … how to treat the patients, and what your infected windows are …’ (Radiographer, Female, Participant 9)

‘[*W*]e did not understand … whether patients were being actually identified as PUIs … We were not getting that information …’ (Radiologist, Female, Participant 6)

### Superordinate theme 3: Personal challenges

Personal challenges experienced by frontline radiology caregivers resulted in three subordinate themes namely: (1) physical health, (2) financial hardships, and (3) social impact. Radiographers complained of burnout because of IPC protocols and increased workload:

‘[*I*]t was tiring when we had the waves … because of the pressure of dressing up, dressing down [*donning and doffing*] … and ensuring the machine and everything was wiped regularly.’ (Radiographer, Female, Participant 11)

‘I got called in the morning, for four COVID-19 mobile x-rays … by the time I had driven to work, I had got a call for 16 … it was exhausting …’ (Radiographer, Female, Participant 10)

Some participants unfortunately contracted COVID-19 and also developed long-term effects of COVID-19 resulting in chronic fatigue:

‘The major implication was contracting COVID-19 and since then I have not been a hundred percent … I get tired quite frequently than I used to.’ (Radiographer, Male, Participant 7)

The decline in the number of patients attending private radiology departments resulted in salary reductions of up to 50% and non-renewal of contracts among private radiographers. Some radiology health caregivers were personally responsible for costs related to COVID-19 management:

‘[*S*]alaries were cut by up to 50% … for those whose contracts were up for renewal, they were completely not taken up … We were working call, driving out multiple times in the night on a reduced salary and not getting paid.’ (Radiographer, Female, Participant 10)

‘[*T*]he medical aid will have certain things that they will not cover, and you have to buy them yourself ….’ (Radiologist, Male, Participant 1)

In as much as respondents were scared of contracting the virus themselves, they were even more worried that they were going to carry the disease home and pose a danger to their immediate families as they felt they had a social shielding responsibility for family members. In the process, interactions with family members were strained:

‘[*B*]ecause we work with COVID-19 patients, I do not want to visit my parents … because they are compromised in terms of their co-morbidities.’ (Radiographer, Female, Participant 11)

## Discussion

Most of the participants did not feel safe while working within their departments during the COVID-19 pandemic because of the perceived unpreparedness of radiology departments in the form of PPE shortages, structural deficiencies limiting proper implementation of IPC protocols, and scarcity of COVID-19- related information; therefore, aligning with studies in other radiology departments (Akudjedu et al. 2021:1219; Murphy et al. 2022:384; Yu et al. 2020:616). Shortage of PPE was attributed to perceived unfair distribution of PPE as radiology and radiography staff were not recognised as frontline workers, echoing the perceived long-standing global issue of non-recognition of radiographers’ well-being and professional contribution to the patient’s healthcare journey (Chevalier et al. 2022:649; Gqweta 2012:24). Together with non-recognition, lack of managerial support presented as perceived barriers to the frontline radiology caregivers’ ability to acquire PPE and the vaccine to protect themselves from COVID-19 as they went about with their duties, which was unique to this study.

Changes to systems and protocols during the pandemic aimed to protect the staff and patients from the virus and aligned with national and international protocols and resonated with experiences in other institutions (Lewis & Mulla 2020:348; Murphy et al. 2022:388); although, social distancing was challenging because of space restrictions. Increased workload, staff shortages, and changes to duties and shift rotation impacted radiographers’ work–life balance. Other studies share similar findings of dearth on information on COVID-19 (McFadden et al. 2022:S19). Incomplete radiology requests missing patients’ COVID-19 status exposed radiographers to the risk of infection and reverberates the ongoing global challenge of incomplete radiology requests, which impact their usefulness (Garba et al. 2021:55). The Royal College of Radiologists (RCR 2021) reiterated the importance of providing adequate clinical background of the patient as part of the responsibilities of referrers.

Social distancing disrupted frontline radiology caregivers’ peer-to-peer relations, which provided social comfort within the workplace. Consequently, this could contribute to employee disengagement, stress, and possibly impact job satisfaction (Mockaitis, Butler & Ojo 2022:1). Consequences of COVID-19 on radiographers’ physical health included contracting the virus, which brought long-lasting effects such as fatigue; therefore, altering participants’ way of life and health. This aligns with systematic reviews and national healthcare systems, which have identified the effects of COVID-19 to exist post-12 weeks of recovery in some individuals and existence of long-COVID-19 (Birman 2023:24; Leung et al. 2020:2190). Use of cassettes for mobile x-rays presented back pain and demonstrates the continued use of computed radiography in some parts of the globe because of its reduced costs of migration from film-screen based radiography in comparison to digital radiography (Bhvita & Cooke 2022).

Considering that the sample comprised participants who are possibly breadwinners, the financial impact of the salary reductions and COVID-19-related medical costs on their families are imaginable. These findings correlate with other studies globally (Itani et al. 2021:5,7; Lewis & Mulla 2020:348; Murphy et al. 2022:388). Family relations were impacted as frontline radiology caregivers avoided interaction with vulnerable family members. They felt they had a social shielding responsibility for protecting their families from possible virus transmission from their workplace; therefore, echoing Murphy et al. (2022:393)’s findings. The administration of contrast media by private radiographers confirms the longstanding challenge of breach of HPCSA scope of practice (Gqweta 2012:24; HPCSA 2020:4). Arguably, extending this role to radiographers as practised in other geographical locations such as the UK could alleviate radiologist shortages that impact service delivery (Bwanga, Kayembe & Sichone 2022:632–633; Koch, Swindon & Pillay 2018:60); however, as far as current policies are concerned, the HPCSA is adamant on the responsibility of cannulation and contrast media administration lying with the radiologists because of potential adverse reactions (HPCSA 2020:4; Koch et al. 2018:34).

### Limitations

Notwithstanding its benefits, the chosen interview-focus group hybrid data-collection method was time consuming as the researchers had to engross themselves in the large amounts of data. To avoid interference with the quality of the study findings, investigator triangulation was adopted as well as repetition of the data analysis steps for each case. Furthermore, incorporating Guba and Lincoln’s Trustworthiness Framework from earlier in the study facilitated trustworthiness of the study findings through reflective journaling and bracketing (Johnson & Christensen 2014).

### Study’s implications

The study has provided baseline information that can be used as part of reflective practice, addressing post-event consequences of COVID-19 among radiology and radiography staff and departments, as well as planning for any future health crises. This includes development programmes to equip managers with skills and knowledge on how to support staff through challenges, continued support of staff suffering from COVID-19 consequences, and development of ways of information sharing among staff and institutions through online means to enable easier coping during future health emergencies. Moreover, the skill and knowledge gaps identified in the study can motivate for further staff developmental training. Among others, this would include educating referring departments on radiology protocols and adequate completion of radiology request forms. The findings have also exposed the poor ventilation and infrastructure within some public radiology departments that requires attention to ensure staff and patient safety.

## Conclusion

Frontline radiology caregivers in the eThekwini district of KZN, South Africa experienced personal and work-related challenges which impacted them physically, financially, and socially. Lack of support from management exacerbated the experiences. It is imperative that solutions, support, and coping mechanisms be developed to assist frontline radiology caregivers and ensure continuity of radiology service delivery during any future health crises.
